# Ameloblastic carcinoma with isolated supramandibular lymph node metastasis in an adolescent: a case report and review of the literature

**DOI:** 10.3389/fonc.2026.1899367

**Published:** 2026-09-24

**Authors:** Yuxin Ma, Chunyao Chen, Ying Li, Wenhui Kong, Linlin Wang

**Affiliations:** 1 Department of Medical Imaging, Jinan Stomatological Hospital, Jinan, Shandong, China; 2 Central Laboratory, Jinan Key Medical and Health Laboratory of Oral Diseases and Tissue Regeneration, Jinan Key Laboratory of Oral Diseases and Tissue Regeneration, Shandong Provincial Key Medical and Health Laboratory of Oral Diseases and Tissue Regeneration, Jinan Stomatological Hospital., Jinan, Shandong, China; 3 School of Stomatology, Binzhou Medical University, Yantai, Shandong, China; 4 Department of Medical Imaging, Jinan City People’s Hospital., Jinan, Shandong, China

**Keywords:** adolescent, ameloblastic carcinoma, CBCT, maxilla, odontogenic tumor

## Abstract

**Objectives:**

To report a rare case of maxillary ameloblastic carcinoma (AC) with buccofacial lymph node metastasis in a 17-year-old male patient, describe its clinical, radiological, and pathological progression, and provide clinical references for routine patient management.

**Case description:**

The patient presented to our hospital with suspected recurrent peripheral ameloblastoma (PA). Cone-beam computed tomography (CBCT) revealed an osteolytic lesion in the right maxilla. Intraoperative frozen section pathology confirmed the diagnosis of ameloblastoma, extended tumorectomy, segmental maxillectomy, and submental island flap reconstruction were conducted. Histopathological examination confirmed the diagnosis of AC. Eight months after segmental maxillectomy, a soft tissue mass overlying the right mandibular buccal region rapidly enlarged. Contrast-enhanced CT revealed heterogeneous enhancement of the lesion with internal necrotic components. Intraoperative pathological examination suggested a malignant tumor. The patient subsequently underwent reoperation: extended resection of the right buccal mass, right suprahyoid lymphadenectomy, and right cervical lymph node dissection. Postoperative pathological examination confirmed AC with supramandibular lymph node metastasis. Following lymph node dissection, the patient received radiotherapy and chemotherapy for two months. At the 6-month follow-up, imaging and clinical examinations revealed no evidence of tumor recurrence or distant metastatic lesions. The patient is currently under regular long-term surveillance to monitor disease progression.

**Conclusion:**

This study reports a case of maxillary AC with isolated supramandibular lymph node metastasis. To our knowledge, this represents the first documented case. Clinicians should be alert to occult buccofacial lymph node metastasis in patients with AC invading the buccal soft tissues, and multimodal imaging is recommended for complete preoperative tumor staging. Individualized combined buccofacial and cervical lymph node dissection may improve locoregional control in patients complicated with buccofacial lymph node metastasis. This study can provide a reference for the identification of rare metastatic patterns and individualized diagnosis and treatment of AC.

## 1 Introduction

AC is a rare malignant odontogenic tumor that exhibits dual histopathological features of both ameloblastoma and carcinoma. It originates from the dental lamina of the oral mucosa and/or mucosal epithelial remnants, and is accompanied by marked cytological atypia, such as nuclear pleomorphism and an elevated nuclear-to-cytoplasmic ratio (1, 2). AC most commonly metastasizes to the lungs and cervical lymph nodes. Metastasis to buccofacial lymph nodes is extremely rare, and no such cases have been previously documented in published literature. Herein, we report a 17-year-old male patient with AC with isolated supramandibular lymph node metastasis. We comprehensively describe the radiological, clinical, and pathological progression from initial presentation to local recurrence, and metastasis.

## 2 Case presentation

### 2.1 Prior treatment at an external hospital

On December 13, 2023, a 16-year-old male patient sought care at an outside hospital due to a gingival mass. Intraoral examination showed a sessile, soft mass measuring approximately 5mm×8mm on the buccal gingiva to teeth 14 and 15. The surface of the mass was erythematous with superficial vascular proliferation. The patient reported intermittent gingival bleeding while brushing teeth, alongside tenderness in the lesion site and widened interdental spaces. CBCT demonstrated an irregular soft tissue mass on the buccal aspect of teeth 14 and 15, with associated buccal alveolar bone resorption. The lesion involved the middle two-thirds of the tooth roots, with no involvement of the maxillary sinus floor (**Supplementary Figure S1**). Intraoperative frozen section pathology confirmed the diagnosis of ameloblastoma. The patient underwent resection of the right maxillary gingival mass and buccal bone plate curettage, with pathological examination confirming peripheral ameloblastoma (**Supplementary Figure S2**).

On 17 May 2024, the patient attended an external hospital with swelling and erosion of the buccal and palatal gingiva in the right maxillary region (teeth 13-17). CBCT revealed the following findings: soft tissue swelling in the surgical area, buccal alveolar bone resorption involving teeth 14-16 with the lesion extending along the entire root length, an expanded range of bone destruction compared with prior images, and thinning of the maxillary sinus floor (**Supplementary Figure S3**).

### 2.2 Initial admission and treatment

On 15 July 2024, the patient was referred to our hospital for further management due to persistent symptoms. Marked gingival inflammation was noted on the distobuccal surfaces of teeth 13–17, manifesting as tissue erosion and bleeding on probing (BOP). The palatal gingiva of teeth 15 and 16 also showed swelling and erosions. Tooth 14 exhibited Grade I mobility. Sensory functions, including lower lip sensation and tongue mobility, were preserved, and no tongue deviation was noted on protrusion. The parotid and submandibular duct orifices appeared normal, without erythema, edema, or abnormal discharge. Neurological examination of buccofacial nerve function and maxillofacial sensory nerves revealed no deficits. Lymph node palpation of the head and neck regions showed no adenopathy, masses, or tenderness. Laboratory tests revealed no abnormalities. The CBCT scan demonstrated bone destruction and resorption on the buccal aspect of teeth 13–17, accompanied by perforation of the maxillary sinus floor (**Supplementary Figure S4**). Recurrence of ameloblastoma was suspected. Intraoperative frozen section pathology confirmed the diagnosis of ameloblastoma. Given that the lesion was a recurrent ameloblastoma with bony destruction spanning approximately 26 mm and involvement of the tooth roots of teeth 13 to 17, extended tumorectomy, segmental maxillectomy, submental island flap transfer and reconstruction, and extraction of teeth 13 to 17 were performed to reduce the risk of recurrence (**Figure 1**).

**Figure 1:**
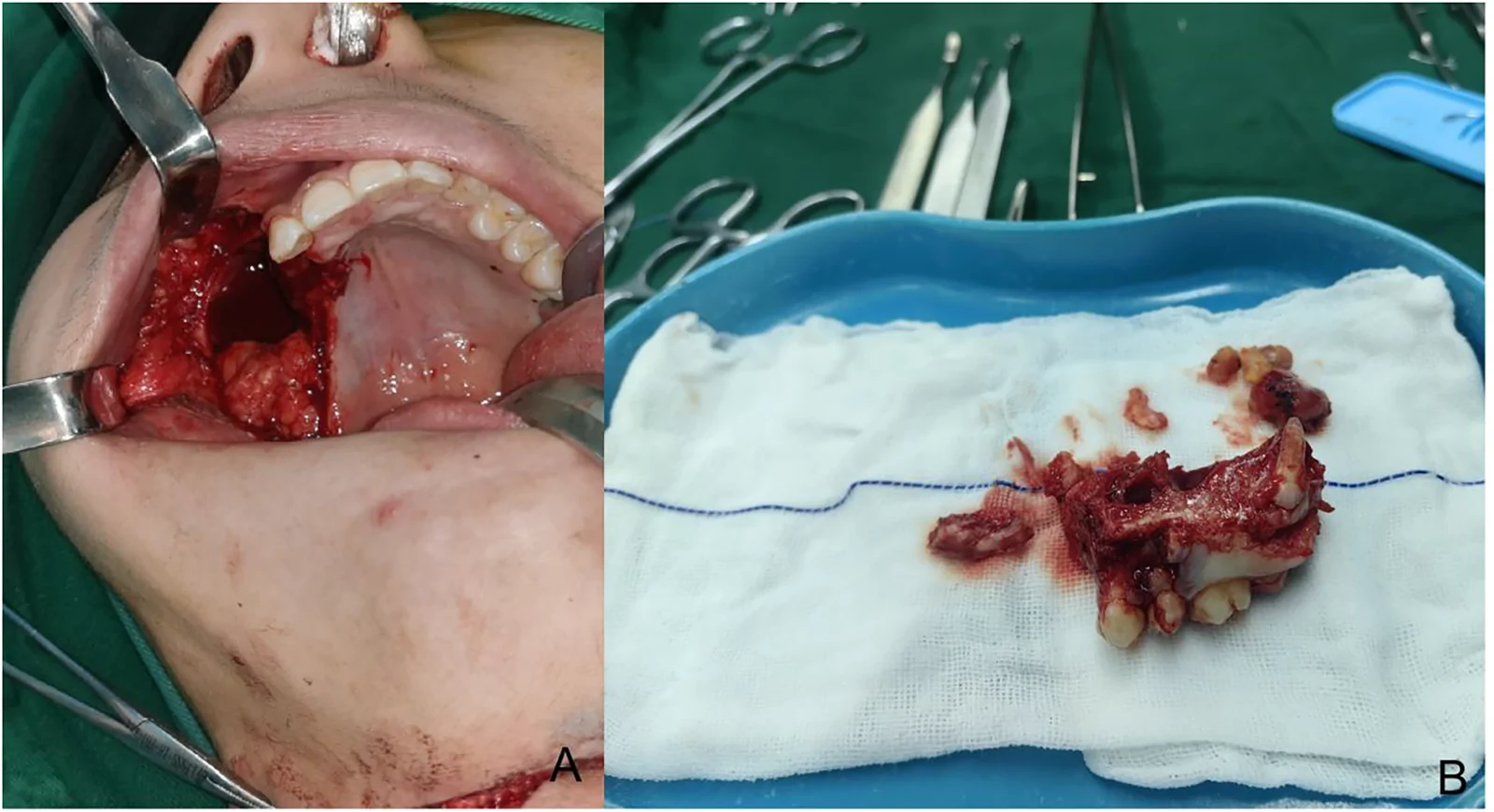
Intraoperative photograph of an extended resection for ameloblastoma of the right maxilla.

Pathological examination revealed an epithelial-derived tumor characterized by diffuse, nested proliferation of carcinoma cells with frequent mitotic figures and local infiltration into the bone marrow cavity (**Supplementary Figure S5**). Immunohistochemical staining showed the following pattern: P40 (+), CK5/6 (+), P53 (~10% positive cells), Ki-67 (~40% proliferative index), CK7 (-), CD31 (no intravascular tumor emboli), D2-40 (no lymphatic invasion), P63 (+), and P16 (+) (**Supplementary Figure S6**). Combining characteristic histomorphology and immunophenotype, the definitive diagnosis of AC was established. Histopathological assessment confirmed negative surgical resection margins with no tumor extension.

Immediate postoperative CBCT examination (24 July 2024) revealed partial absence of the right maxilla and loss of teeth 13–17 (**Supplementary Figures S7A, B**). Four months after the extended resection, follow-up CBCT (17 November 2024) demonstrated satisfactory healing at the surgical site, with no evident bone destruction or soft tissue mass (**Supplementary Figures S7C, D**).

### 2.3 Re-hospitalization and treatment

On 22 March 2025, the patient was readmitted to our hospital for sudden enlargement of a buccal soft tissue mass over the right mandible, with the lesion measuring approximately 23 mm × 19 mm × 16 mm, exhibiting firm texture, moderate mobility, and no tenderness, pulsation, or overlying skin changes. Facial sensation remained intact, and buccal nerve function was unremarkable on clinical examination. Mouth opening was unrestricted with a physiologic trajectory. Bilateral masticatory muscles were non-tender. The teeth 13-17 were missing, and there was a partial maxillary defect on the right side. The reconstructed flap appeared normal in color. A mobile, non-tender lymph node (approximately 1.0 cm in diameter) was palpable in the left submandibular region. A smaller, mobile, and non-tender lymph node (approximately 0.5 cm) was detected on the right submandibular side. Review of previous CBCT: A slight gray-level difference was noted in the soft tissue lateral to the right mandible, measuring about 21 mm × 12 mm × 16 mm (**Supplementary Figures S3E–H**, **S4E–H**). Contrast-enhanced computed tomography (CECT) performed on March 22, 2025 identified a 23 mm × 19 mm × 16 mm soft tissue mass within the right buccal space, with ill-defined margins contiguous with adjacent musculature. The non-contrast scan showed uneven density with patchy hypodense areas centrally (**Figure 2A**). The mass exhibited heterogeneous enhancement on contrast imaging, with moderate enhancement in the solid components and no enhancement in the central necrotic hypodense area (**Figure 2B**). No bone destruction was seen in the adjacent mandible (**Figure 2C**). Postoperative changes were noted in the right maxilla (**Figure 2D**). Intraoperative frozen section histopathology revealed malignant epithelial lesions. Under general anesthesia, radical surgical intervention was performed, including extended resection of metastatic carcinoma of the right buccal soft tissue, right suprahyoid lymphadenectomy, and right cervical lymph node dissection.

**Figure 2:**
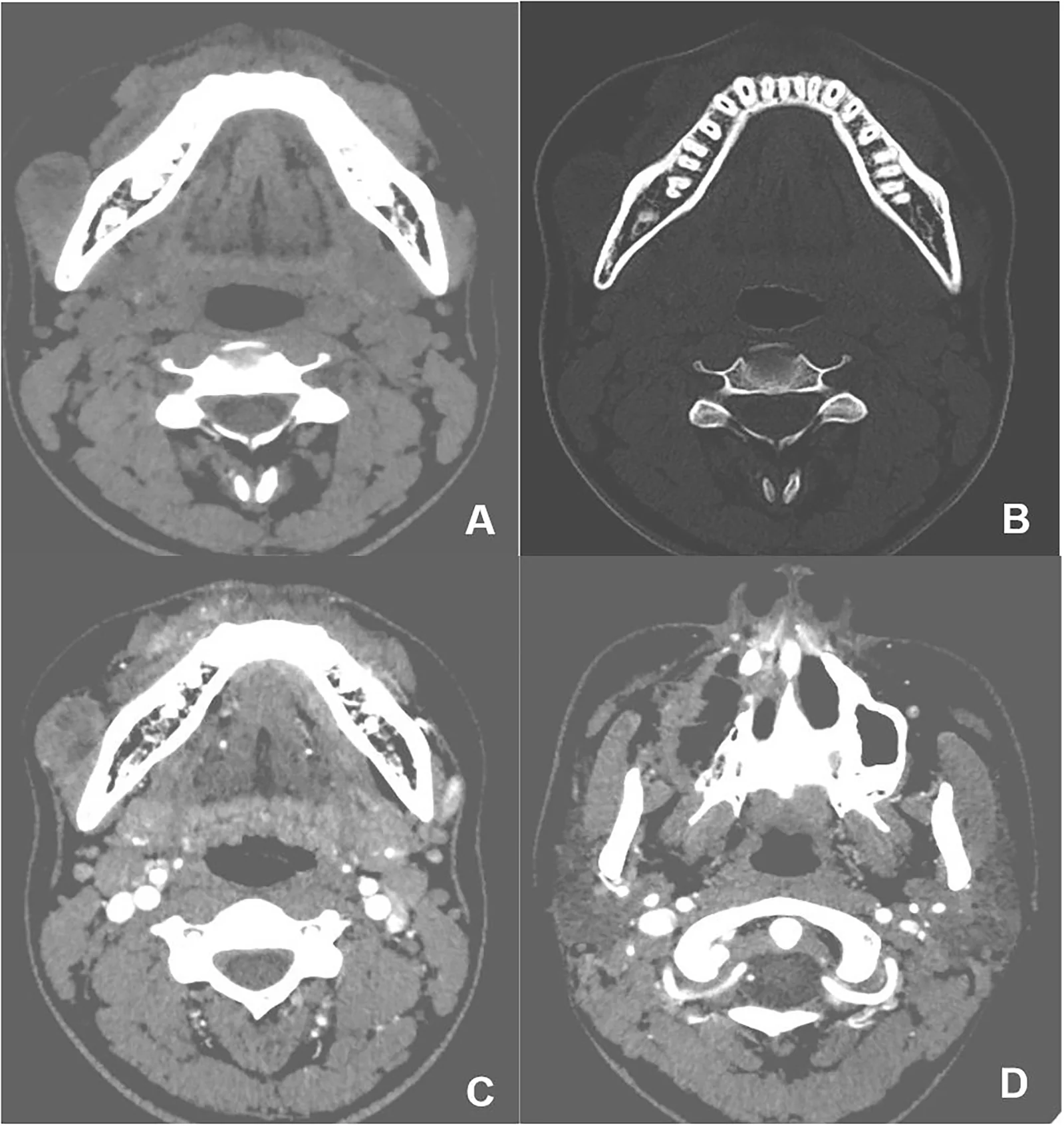
CT examination (22 March 2025). **(A)** Axial CT plain scan shows a soft tissue mass in the right buccal region. **(B)** Axial enhanced CT scan reveals uneven enhancement of the lesion with visible necrotic areas. **(C)** Axial bone window shows no obvious destruction of the surrounding bone. **(D)** Axial enhanced CT scan shows no soft tissue mass in the surgical area.

Histopathological analysis confirmed supramandibular lymph node metastasis with infiltration of perilesional striated muscle. Metastatic carcinoma was absent in all 12 harvested right cervical and submandibular lymph nodes (**Figure 3**).

**Figure 3:**
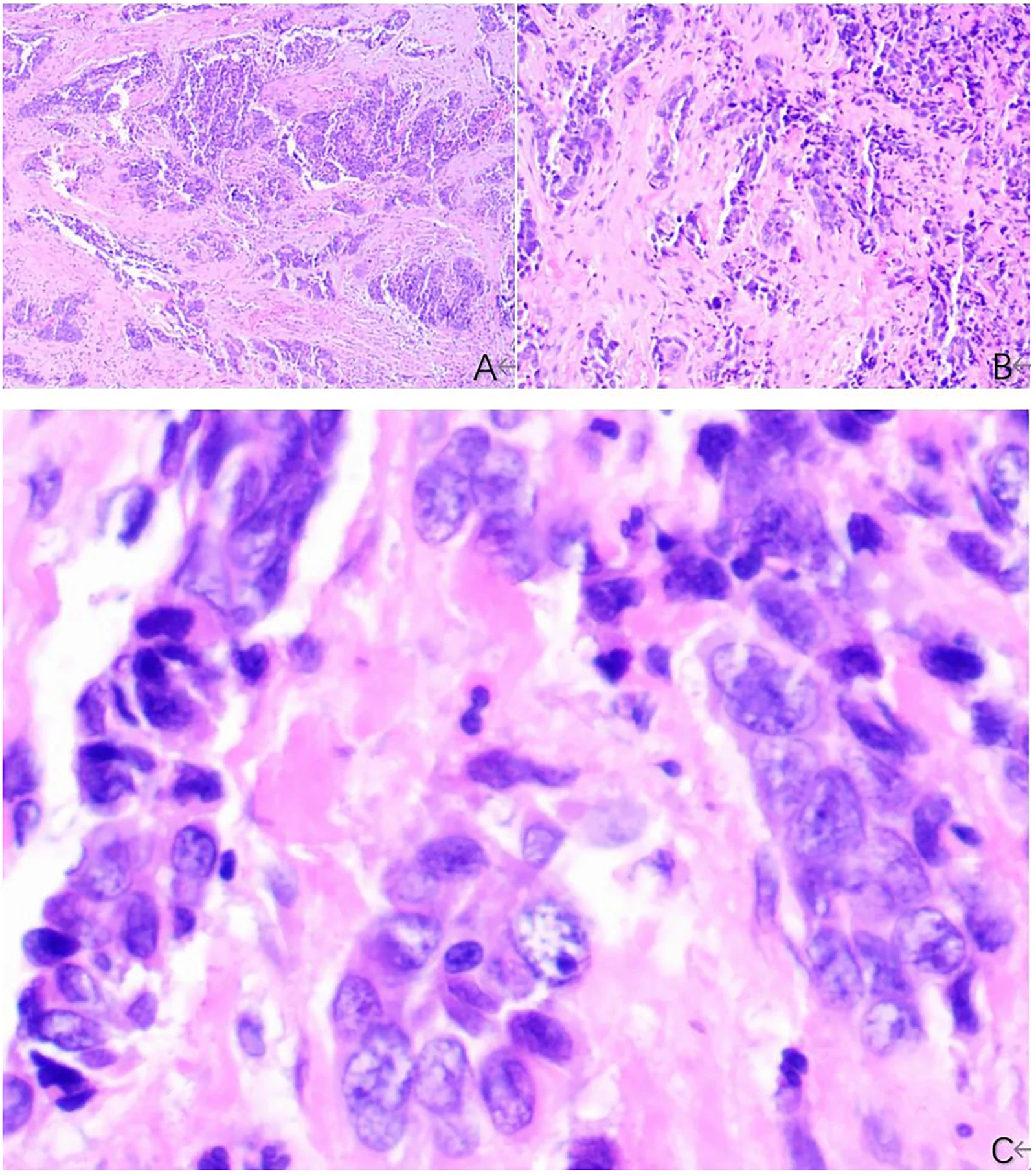
Light microscopic findings. The pathological examination confirms a diagnosis of AC. **(A)** The tumor exhibits irregular, sheet-like, nested, and reticular patterns of infiltrative growth with ill-defined borders, infiltrating into the surrounding bone and/or fibrous connective tissues. **(B)** The tumor cells are densely packed with pleomorphic nuclei of varying sizes. Abnormal, triphasic, and multipolar mitotic images are frequently observed. **(C)** The cells exhibit marked atypia, an increased nuclear-to-cytoplasmic ratio, irregular nuclear membranes, coarse chromatin, prominent nucleoli, and pleomorphic morphology. **(A)** (HE, 40×), **(B)** (HE, 100×), **(C)** (HE, 400×).

Immunohistochemistry: P40 (+), CK5/6 (+), EGFR (+), Ki-67 (~50% positive cells), CD99 (+), P16 (+), NUT (-), NKX2.2 (-) (**Figure 4**). Combined histomorphologic and immunohistochemical findings confirmed isolated supramandibular lymph node metastasis arising from AC.

**Figure 4:**
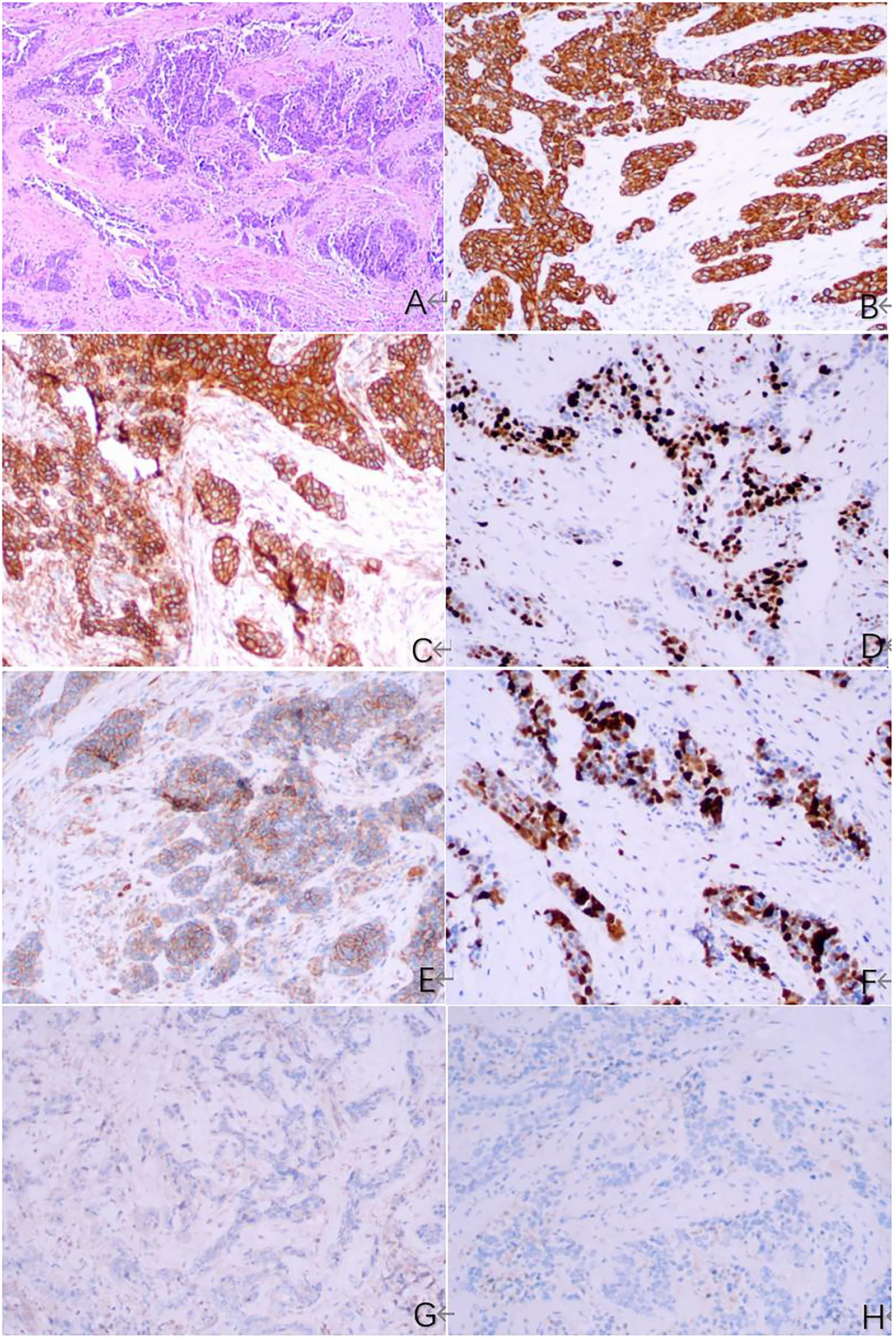
Immunohistochemistry. **(A)** P40 (+), **(B)** CK5/6 (+), **(C)** EGFR (+), **(D)** Ki-67 (~50% positive cells), **(E)** CD99 (+), **(F)** P16 (+), **(G)** NUT (-), **(H)** NKX2.2 (-).

### 2.4 Adjuvant concurrent chemoradiotherapy

The patient underwent extended resection for metastatic carcinoma of the right oral cavity with an uneventful postoperative course and well-healed surgical incision. After suture removal, the patient was transferred to another hospital for adjuvant postoperative chemoradiotherapy to eliminate potential occult micrometastases and reduce the risk of tumor recurrence. The initial radiotherapy target volume covered the primary tumor bed. The clinical target volume (CTV) was defined as the gross tumor volume (GTV) with a 1 cm margin, level Ia, bilateral cervical levels Ib, II, III, and right-sided cervical levels IVa and IX, with a prescribed dose of 50 Gy delivered in 25 fractions. Subsequently, field reduction was performed by excluding the right cervical level IVa, and a booster dose was administered to a total of 60 Gy in 30 fractions. A further field reduction was targeted at the base of the metastatic buccofacial lymph nodes on the right side, with an additional boost dose up to 66 Gy in 33 fractions. Concurrent cisplatin-based chemotherapy was administered throughout the radiotherapy course due to extracapsular extension of the metastatic lymph nodes. Treatment-related adverse events included grade I oral mucositis and transient myelosuppression, both of which resolved completely after symptomatic supportive treatment.

### 2.5 Post-therapy follow-up outcome

Follow-up magnetic resonance imaging (MRI) and CT examinations performed on September 22, 2025, revealed no evidence of local tumor recurrence, residual disease, or distant metastasis (**Supplementary Figure S8**). The patient currently maintains a good general condition and satisfactory quality of life. Regular telephone follow-up has detected no signs of local recurrence or systemic distant metastasis to date.

This study was approved by the Medical Ethics Committee of Jinan Stomatological Hospital (JNSKQYY-2025-012), and the patient and the patient’s family provided informed consent.

## 3 Discussion

### 3.1 Epidemiological and clinical features of maxillary AC in adolescents

AC represents an exceptionally rare malignant odontogenic tumor, accounting for <1% of all head and neck malignancies. In early classifications, it was categorized into primary and secondary types, but according to the 2017 WHO classification, this tumor is no longer separated into primary and secondary types and is now recognized as a single entity (3), a classification that is still in use today. The 2024 WHO Classification of Head and Neck Tumors recognizes four primary ameloblastoma subtypes: conventional, unicystic, peripheral, and metastasizing. The peripheral subtype accounts for roughly 2% of all ameloblastoma diagnoses, making it a rare variant (4). PA is a benign odontogenic epithelial tumor arising within extraosseous jaw soft tissues, with primary involvement of the gingiva and alveolar mucosa. Combined with the patient’s prior medical records from an external hospital, this case was clinically suspected to represent malignant transformation of PA into AC. Li et al. (5) reviewed 538 patients with ameloblastoma and found merely 12 cases (2.2%) progressed to malignant AC. Philipsen’s cohort study (6) of 160 PA cases demonstrated only six lesions with malignant biological potential. Therefore, malignant transformation of peripheral ameloblastoma into AC is an exceedingly rare event. Clinically, PA manifests as a well-circumscribed exophytic mass that may compress the jaw cortex and induce superficial saucer-like bone resorption. The primary lesion in our patient originated as an exophytic buccal gingival mass that invaded the buccal alveolar bone plate. Preoperative imaging delineated a distinct border separating the gingival mass from adjacent bone, with bone resorption displaying an outside compressive pattern-radiological features consistent with PA. Intraoperative frozen pathology from the outside hospital initially suggested ameloblastoma. Unfortunately, the original paraffin blocks were unavailable for additional immunohistochemical staining, which precluded comprehensive assessment of cellular atypia. Accordingly, definitive differentiation between primary and secondary lesions could not be achieved in this patient. AC demonstrates a male predominance, with a male-to-female ratio of 2:1. The tumor occurs across a wide age range (4-90 years), with higher prevalence among middle-aged and older adult patients. According to Chen et al. (7), only 80 maxillary AC cases were reported between 1948 and 2021, of which just 10 occurred in adolescents aged 7-18 years. The posterior mandible is the most common site, comprising approximately two-thirds of cases, while maxillary involvement is less frequent (8, 9).

The typical clinical symptoms of AC include jaw swelling, pain, buccofacial asymmetry, trismus, gingival swelling or ulceration, tooth mobility or loss, pathological fractures, and neurological dysfunction in the affected area. Because of its high invasiveness, AC frequently involves surrounding organs (such as the nasal cavity and orbit), leading to additional symptoms including excessive tearing, nasal obstruction, globe displacement, and visual impairment (9–11). The differential diagnosis of ameloblastoma and AC is clinically challenging. AC presents with paresthesia, rapid growth, cortical bone perforation, and pain.

### 3.2 Metastatic patterns of AC

AC exhibits three metastatic patterns: local direct invasion, lymphatic metastasis, and hematogenous metastasis. In a literature review by Kruse et al. (8), pulmonary metastasis was documented in 26.9% of AC cases. According to Wei X et al. (12), distant metastasis occurred in 11 of 98 patients with AC: 4 cases had isolated pulmonary metastasis, 4 had cervical lymph nodes metastasis, 1 had hepatic metastasis, and 2 presented with extensive metastasis involving the lungs, pleura, and bone marrow. Hoehnle et al. (11) reported one case of AC with hepatic metastases. Zwahlen et al. (13) documented a case of malignant ameloblastoma of the jaw with cardiac metastasis. Harada et al. (14) reported a case of AC with vertebral metastasis. The most common lymphatic metastatic pattern of AC follows a sequential cervical lymphatic drainage pathway. After eroding the buccal cortical bone, the tumor spreads along the superficial vestibular lymphatic vessels and sequentially involves the submandibular and superior deep cervical lymph nodes. Buccofacial lymph nodes are anatomically categorized into four groups: zygomatic, infraorbital, buccal, and supramandibular nodes. These lymph nodes are distributed within the subcutaneous space along buccofacial vascular branches and maintain a close anatomical adjacency with the buccofacial artery and vein. The present case reports an AC with rare supramandibular lymph node metastasis. Supramandibular lymph nodes are located at the anterior border of the masseter muscle or at the attachment site of the buccinator muscle to the mandible. Metastasis to supramandibular lymph nodes is predominantly observed in lower gingival carcinoma and, secondarily, in buccal mucosal carcinoma. This lymphatic basin receives lymphatic drainage from the upper buccofacial region, lower lip, and entire buccal soft tissue, with efferent lymphatic vessels primarily draining into the submandibular lymph nodes (15). Based on the anatomical drainage characteristics and clinical course of this case, we hypothesize that the supramandibular lymph nodes represented the first-station draining lymph node for lymphatic metastasis in this patient.

A retrospective review of preoperative imaging confirmed supramandibular lymphadenopathy was already present prior to the initial July 2024 surgery. From a clinical optimization perspective, preoperative comprehensive evaluation of soft tissue and lymph node status using CECT or MRI, combined with intraoperative extended resection of suspicious invasive soft tissues, could effectively minimize the risk of further tumor dissemination. The failure to identify the supramandibular lymph node metastasis preoperatively was attributable to a combination of objective limitations and insufficient recognition of this rare metastatic pattern in previous clinical practice. The key restrictive factors are summarized as follows. First, inaccurate preoperative prediction of tumor biological behavior. Based on the patient’s surgical history at an external hospital and intraoperative frozen section results, recurrent ameloblastoma was initially suspected; the patient’s guardians declined CT over concerns of excessive radiation exposure in the adolescent. Second, inherent limitations in preoperative imaging evaluation. Influenced by the presumptive benign diagnosis, only CBCT was performed to evaluate bony destruction. Meanwhile, the patient’s guardians declined contrast-enhanced CT examination due to concerns regarding radiation exposure in the adolescent patient. CBCT provideed superior visualization of bony structures but fails to identify tiny lymph nodes. In addition, no enlarged lymph nodes were detected on physical examination, yielding no positive clinical clues for occult lymphatic metastasis. Third, difficult differential diagnosis of maxillofacial soft-tissue lesions in adolescents. Inflammatory soft-tissue hyperplasia is prevalent in the adolescent buccofacial region. The patient was asymptomatic at the early disease stage, making subtle soft-tissue abnormalities undetectable before rapid tumor enlargement prompted medical consultation. Accordingly, the possibility of early lymphatic invasion and micrometastasis was not recognized preoperatively. Fourth, insufficient clinical evidence and standardized protocols. To date, no cases of AC with isolated supramandibular lymph node metastasis have been documented in the literature, and there is no standardized clinical protocol for routine screening of supramandibular lymph node for this disease.

To our knowledge, this article reports the first documented case of AC presenting with supramandibular lymph node metastasis. The findings of this study provide evidence-based insights for the standardized diagnosis and management of buccofacial lymph node metastasis in AC, improve clinical awareness of this rare metastatic pattern, and reduce the risk of missed diagnosis of occult buccofacial lymph node metastasis.

### 3.3 Histopathological and immunohistochemical characteristics

Histologically, AC is morphologically similar to ameloblastoma. Tumor islands and cords of AC feature peripheral palisaded columnar cells with central stellate reticulum cells. In addition, AC has obvious cytological malignant characteristics, that is, most of the central stellate reticulum cells have disappeared and are replaced by poorly differentiated spindle epithelial cells, oval or short spindle basal cells, and the tumor cells are characterized by pleomorphism, hyperchromatic nuclei and increased mitotic figures. This case suggests that intraoperative frozen section pathology is limited by sampling bias and morphological evaluation, which may underestimate the malignant potential of tumors. Postoperative immunohistochemical review is critical for identifying cellular atypia. Microscopically, the tumor in this case showed obvious cellular pleomorphism and mitotic figures, with local invasive growth invading the surrounding bone and soft tissues. Focal squamous epithelial differentiation and metaplasia were observed in the lesion, with disordered cell arrangement and loss of polarity, which was consistent with the microscopic morphological characteristics of AC. The expression level of Ki-67 in AC is significantly higher than that in ameloblastoma, and the high expression of Ki-67 is closely related to the invasiveness and recurrence risk of AC (16, 17). The immunohistochemical results of this case showed that CK19 was positive and CK7 was negative, which supported the odontogenic epithelial origin of the tumor and excluded the origin of ordinary adenocarcinoma; CK5/6, P40 and P63 were all positive, suggesting that the tumor was accompanied by squamous epithelial differentiation, which was consistent with the immunophenotypic characteristics of AC with squamous metaplasia. In addition, the Ki-67 positive proliferation index was about 40%, indicating that the tumor had moderate proliferative activity, active cell growth and certain malignant invasive potential. P16 overexpression is widely recognized as a surrogate marker for HPV-associated oropharyngeal squamous cell carcinoma; however, the present case involved an odontogenic tumor rather than a mucosal squamous cell carcinoma. Nevertheless, combined with the negative NUT staining result, P16 upregulation in this case was primarily attributed to aberrant tumor cell cycle dysregulation. Epidermal growth factor receptor (EGFR) was immunohistochemically positive in this case. EGFR acts as a key driver of local invasiveness in ameloblastoma, and its overexpression is strongly correlated with tumor infiltration into adjacent bone tissue. Positive CD99 expression reduces intercellular adhesion of tumor cells, facilitates local infiltration, and indicates the presence of potential microscopic invasive foci, which may lead to diagnostic confusion with tumors such as Ewing sarcoma. Ewing sarcoma shows diffuse strong CD99 immunoreactivity and nuclear NKX2.2 positivity, and co-positivity of the two markers represents its core diagnostic indicator, while NKX2.2 is absent in AC (18). Furthermore, combined analysis of histomorphology and immunohistochemistry markers enables complete differentiation from Ewing sarcoma, NUT carcinoma and mucosal squamous cell carcinoma.

### 3.4 Imaging evaluation strategy for AC

Ameloblastoma and AC are typically evaluated using CBCT, CT, MRI, and panoramic radiographs. Panoramic radiographs are often used as a preliminary screening tool and can reveal nonspecific changes such as jaw expansion and root resorption. CT and CBCT are particularly important for assessing the extent of bone destruction, cortical perforation, and internal tumor architecture (e.g., multilocular or unilocular) (19). MRI can be used to observe the signal characteristics within the lesion and the extent of tumor infiltration, particularly when both solid and mixed cystic features are present (12, 20). Moreover, in cases with metastasis (such as to the lung or lymph nodes), PET-CT usually reveals hypermetabolic lesions (7, 11). Previous multiple imaging examinations of this case have demonstrated that CBCT has significant advantages in evaluating bone destruction, but it has limitations such as a small field of view and low soft tissue resolution. The combined application of multiple imaging techniques is the optimal strategy for evaluating ameloblastoma, AC, and metastatic lesions. Imaging manifestations of ameloblastoma and AC are similar. Both present as unilocular or multilocular radiolucent lesions. However, ameloblastoma tends to have relatively well-defined regular borders, while AC manifests obvious aggressive features (21). AC manifests radiologically as a mass invading adjacent osseous and soft tissue structures, and often exhibits a “moth-eaten” or infiltrative growth pattern features (7, 10). In some cases of AC, there is a mixed pattern of radiolucency and radiopacity, indicating the coexistence of osteolytic and osteogenic destruction within the tumor (22). Such mixed-density features are less commonly seen in benign ameloblastoma and occur more frequently in cases undergoing malignant transformation. In addition, multilocular (pseudounilocular) ameloblastomas are more likely to exhibit malignant features, with central necrotic areas being more common and heterogeneous enhancement frequently seen on contrast-enhanced imaging (11, 20). In addition, AC is characterized by rapid growth and often demonstrates a marked increase in volume (23). The CBCT images showed a rapidly progressive area of bone destruction with ill-defined margins and surrounding soft tissue swelling. The imaging features of this case are highly consistent with the typical growth pattern of AC.

### 3.5 Differential diagnosis of AC

AC requires differentiation from PA, central ameloblastoma, maxillofacial osteomyelitis, gingival squamous cell carcinoma, and soft tissue sarcomas.

1. PA: PA is a benign odontogenic epithelial lesion characterized by slow growth, mild compressive bone resorption, absent cellular atypia, and extremely rare metastasis. The present case exhibited rapid tumor enlargement, marked nuclear pleomorphism, and buccofacial lymph node metastasis, which excluded a diagnosis of simple PA.
2. Central Ameloblastoma: This tumor originates intraosseously and typically presents as a multilocular radiolucent bony destruction lesion. In contrast, the primary lesion in this case arose from gingival soft tissue, consistent with an extraosseous origin.
3. Osteomyelitis: Osteomyelitis is radiologically characterized by sequestrum formation and periosteal reaction, with no atypical neoplastic epithelial proliferation observed histologically (24). The patient presented without fever, purulent discharge, or typical inflammatory bony sequestra, which ruled out inflammatory osteomyelitis.
4. Gingival Squamous Cell Carcinoma: Gingival squamous cell carcinoma is generally positive for CK7 and negative for odontogenic epithelial markers (P40/CK5/6). The tumor in this case displayed positive expression of odontogenic markers, thereby excluding mucosal squamous cell carcinoma.
5. Soft Tissue Sarcoma: Although positive CD99 expression may be detected in certain sarcomas, the tumor was negative for NUT and NKX2.2. Combined with the odontogenic epithelial morphological features of the lesion, a soft tissue sarcoma was definitively excluded.

### 3.6 Individualized surgical and adjuvant treatment principles

The preferred treatment for PA is local extended resection. If the tumor involves the periosteum, the tumor and the involved periosteum should be completely resected; for those with obvious compression of the cortical bone, a thin layer of 1–2 mm cortical bone can be ground off. If the involved teeth have root resorption, loosening and other conditions, the involved teeth should be extracted and the periodontal membrane should be curetted. If residual tumor or recurrence is detected, marginal osteotomy should be routinely performed (25). Continuous follow-up is necessary due to the possibility of recurrence or malignant changes.

The therapeutic strategy for AC is primarily based on its biological behavior and potential risk of metastasis. Surgery is the mainstay of treatment for AC, usually involving extended tumor resection or partial jaw resection to achieve adequate negative margins (26). For large tumors or those invading critical structures, partial jaw resection combined with immediate reconstruction (such as free flap transplantation) may be required to restore function and aesthetics (27). The lesion involved teeth 13 to 17 of the right maxilla, with perforation of the maxillary sinus floor. The primary clinical objective was radical resection of the recurrent lesion to achieve negative surgical margins. Therefore, segmental maxillectomy combined with submental island flap transfer and reconstruction was performed. Segmental maxillectomy with tumor-free bony margins allows complete eradication of microinfiltrative lesions, while flap reconstruction effectively prevents postoperative oronasal fistula and restores normal oral function. Prophylactic cervical lymph node dissection is not routinely recommended for patients with AC (28). Cervical lymphadenectomy is only indicated in cases with pathologically confirmed cervical lymph node metastasis. The inferior border of the mandible serves as the caudal boundary of standard cervical lymph node dissection; thus, conventional cervical dissection compartments fail to include the buccofacial lymph node basin. For patients presenting with buccofacial lymphatic spread, isolated cervical lymphadenectomy alone is inadequate. Accordingly, combined buccofacial-cervical lymph node dissection may be required in selected patients with confirmed buccofacial lymphatic spread. In terms of adjuvant therapy, radiotherapy and chemotherapy have limited efficacy in treating localized AC. However, in cases of metastasis, chemotherapy remains the only available treatment option. Radiotherapy can be used in cases with residual lesions postoperatively, particularly in patients who are inoperable or unsuitable for repeat surgery (24). Given the high proliferative activity of the tumor (Ki-67 index ≈ 40%), extracapsular lymph node invasion and high risk of occult micrometastases, postoperative concurrent chemoradiotherapy was administered. Radiation therapy targets and eliminates residual local tumor cells, whereas cisplatin acts as a radiosensitizer to improve local tumor control and reduce the risk of distant metastasis.

### 3.7 Study limitations

This case report has several notable limitations. First, the post-chemoradiotherapy follow-up duration is only 6 months. Long-term surveillance over 3–5 years is still ongoing, and late recurrence or distant metastasis risk cannot be fully evaluated at present. Second, the original pathological wax blocks of the initial operation in the external hospital were unavailable, so secondary immunohistochemical verification of the primary benign lesion could not be performed, limiting the judgment of malignant transformation origin.

## 4 Patient’s perspective

Following initial maxillary resection and flap reconstruction, he reported mild difficulty with chewing and mild buccofacial asymmetry, which resulted in short-term poor self-esteem. During postoperative radiotherapy and chemotherapy, he experienced mild oral mucositis and fatigue, but maintained good treatment compliance with psychological guidance from medical staff and family members. The patient and his guardians expressed major concerns about tumor metastasis risk before the second reoperation; they fully accepted radical resection and lymph node dissection after detailed communication of the aggressive biological behavior of AC. At the 6-month follow-up, the patient reported satisfactory quality of life, no persistent pain, and returned to normal study and daily social activities. The family emphasized that regular long-term imaging follow-up is essential for early detection of recurrent lesions in adolescents with malignant odontogenic tumors.

## 5 Clinical recommendations

This study reports an adolescent case of maxillary AC accompanied by buccofacial lymph node metastasis, and we put forward several individualized clinical management recommendations based on this case. Intraoperative frozen pathology is prone to underestimating the malignant phenotype and metastatic potential of aggressive or recurrent ameloblastic lesions, attributed to insufficient sampling and morphological artifacts generated during frozen section processing. Postoperative immunohistochemistry to identify cellular atypia is thus indispensable for revising and finalizing the pathological diagnosis. Once a diagnosis of AC is established, multimodal imaging is required to complete preoperative tumor staging evaluation. Clinicians should maintain a high index of suspicion for occult buccofacial lymph node metastasis in patients with AC invading the buccal soft tissue. For patients with confirmed buccofacial lymphatic metastasis, individualized combined buccofacial and cervical lymph node dissection can decrease the risks of regional tumor residue and local recurrence.

## Data Availability

The original contributions presented in the study are included in the article/**Supplementary Material**. Further inquiries can be directed to the corresponding author.

## References

[1] Osama MA Rao S Kunvar S Koul RK Nanda BS . Transformation of ameloblastoma to ameloblastic carcinoma in a 10-year-old child. J Cancer Res Ther. (2023) 19:S0. doi: 10.4103/jcrt.jcrt_282_22 37147971

[2] John S Ramya S Verma S Samadi FM Gupta S . Clear and spindle cell dedifferentiation in ameloblastic carcinoma: A case report on a uncommon phenomenon. Indian J Otolaryngol Head Neck Surg. (2024) 76:1229–33. doi: 10.1007/s12070-023-04232-z 38440659 PMC10908652

[3] Vered M Wright JM . Update from the 5th edition of the World Health Organization classification of head and neck tumors: Odontogenic and maxillobuccofacial bone tumours. Head Neck Pathol. (2022) 16:63–75. doi: 10.1007/s12105-021-01404-7 35312978 PMC9019005

[4] World Health Organization International Agency for Research on Cancer . WHO classification of tumours. In: Head and Neck Tumours, 5th ed, vol. 9. Lyon: International Agency for Research on Cancer (2024). p. 339–46.

[5] Li J Du H Li P Zhang J Tian W Tang W . Ameloblastic carcinoma: An analysis of 12 cases with a review of the literature. Oncol Lett. (2014) 8:914–20. doi: 10.3892/ol.2014.2230 25013517 PMC4081393

[6] Philipsen HP Reichart PA Nikai H Takata T Kudo Y . Peripheral ameloblastoma: Biological profile based on 160 cases from the literature. Oral Oncol. (2001) 37:17–27. doi: 10.1016/s1368-8375(00)00064-6 11120479

[7] Chen IY Giampoli EJ Zhang D . Ameloblastic carcinoma of the maxilla: A rare case report and review of literature from 1948 to 2021. Int J Surg Pathol. (2023) 31:442–54. doi: 10.1177/10668969221102542 35668625

[8] Kruse AL Zwahlen RA Grätz KW . New classification of maxillary ameloblastic carcinoma based on an evidence-based literature review over the last 60 years. Head Neck Oncol. (2009) 1:31. doi: 10.1186/1758-3284-1-31 19674470 PMC2733136

[9] Khan F Somawardana I Mitchell M Adewumi MT Berger B Marmie B . Ameloblastic carcinoma of the maxilla with uncommon sinus manifestations. Ear Nose Throat J. (2024). doi: 10.1177/01455613241245207 38561954

[10] Barca I Ferragina F Kallaverja E Cristofaro MG . A rare case of secondary ameloblastic carcinoma in a young man. Ann Ital Chir. (2023) 93:S2239253X23039385. Published 2023 Apr 18. 37199484

[11] Hoehnle NS Beutler BD Ulanja MB Rastegarpour A . Ameloblastic carcinoma with hepatic metastases: A case report and review of ameloblastic carcinoma. J Clin Imaging Sci. (2022) 12:58. doi: 10.25259/JCIS_115_2022 36601607. Published 2022 Oct 27. PMC9805602

[12] Wei X . A case report and literature review of secondary ameloblastic carcinoma of the mandible. Changchun: Jilin University (2022). doi: 10.27162/d.cnki.gjlin.2022.005613

[13] Zwahlen RA Vogt P Fischer FS Grätz KW . Case report: Myocardial metastasis of a maxillary malignant ameloblastoma. J Oral Maxillofac Surg. (2003) 61:731–4. doi: 10.1053/joms.2003.50146 12796889

[14] Harada H Tanaka A Kimura M Ieda S Wada T Matsumura T . Spindle cell variant of ameloblastic carcinoma: Another example in a Japanese male. Case Rep Dent. (2023) 2023:8755637. doi: 10.1155/2023/8755637 36970563. Published 2023 Mar 16. PMC10036176

[15] Tang X Zhong YH . Significance of buccinator and supramandibular lymph node metastasis after surgery for buccal mucosal carcinoma. J Wuhan Univ (Med Sci). (2026) 47:1–5. doi: 10.14188/j.1671-8852.2026.0381

[16] Marín-Márquez C Adisa AO Niklander SE Kirby J Hunter KD . Genomic and transcriptomic analysis of ameloblastoma reveals distinct molecularly aggressive phenotypes. Mod Pathol. (2025) 38:100682. doi: 10.1016/j.modpat.2024.100682 39675431

[17] Liu XH Zhong NN Yi JR Lin H Liu B Man QW . Trends in research of odontogenic keratocyst and ameloblastoma. J Dent Res. (2025) 104:347–68. doi: 10.1177/00220345241282256 39876078

[18] Bal M Shah A Rekhi B Mittal N Rane SU Rabade K . Adamantinoma-like Ewing sarcoma of the head and neck: A case-series of a rare and challenging diagnosis. Head Neck Pathol. (2022) 16:679–94. doi: 10.1007/s12105-022-01412-1 35025056 PMC9424385

[19] Kim JE Cho JB Yi WJ Heo MS Lee SS Huh KH . Classification and prognostic evaluation of ameloblastoma using multiplanar CT imaging: A retrospective analysis. BMC Oral Health. (2025) 25:115. doi: 10.1186/s12903-025-05485-6 39844117. Published 2025 Jan 22. PMC11755854

[20] Gomes JPP Ogawa CM Silveira RV Castellano G De Rosa CS Yasuda CL . Magnetic resonance imaging texture analysis to differentiate ameloblastoma from odontogenic keratocyst. Sci Rep. (2022) 12:20047. doi: 10.1038/s41598-022-20802-7 36414657. Published 2022 Nov 21. PMC9681845

[21] Song Y Ma S Mao B Xu K Liu Y Ma J . Application of machine learning in the preoperative radiomic diagnosis of ameloblastoma and odontogenic keratocyst based on cone-beam CT. Dentomaxillofac Radiol. (2024) 53:316–24. doi: 10.1093/dmfr/twae016 38627247 PMC11211686

[22] Deorah DS Gupta DS . Exploring the rarity: Clinical insights and histopathological diversity of hybrid desmoplastic-plexiform ameloblastoma. Semin Diagn Pathol. (2025) 42:22–4. doi: 10.1053/j.semdp.2024.12.001 39706779

[23] Smit C Robinson L van Heerden MB Meyer PW . Radiologic specific growth rate of ameloblastomas: A clinicopathological correlation. J Oral Pathol Med. (2025) 54:161–72. doi: 10.1111/jop.13611 39887743 PMC11891031

[24] Ghai S . Ameloblastoma: An updated narrative review of an enigmatic tumor. Cureus. (2022) 14:e27734. doi: 10.7759/cureus.27734 36127985. Published 2022 Aug 6. PMC9481193

[25] Zhang X Tian X Hu Y Zhang C Wei C Yang X . Oral peripheral ameloblastoma: A retrospective series study of 25 cases. Med Oral Patol Oral Y Cirugía Bucal. (2018) 23:e277–81. doi: 10.4317/medoral.22225 29680843 PMC5945233

[26] Alarcón-Sánchez MA Becerra-Ruíz JS Yessayan L Mosaddad SA Heboyan A . Implant-supported prosthetic rehabilitation after Ameloblastomas treatment: A systematic review. BMC Oral Health. (2023) 23:1013. doi: 10.1186/s12903-023-03765-7 38110933. Published 2023 Dec 18. PMC10729555

[27] Al Omari F Hakami RM . Multidisciplinary approach to mandibular ameloblastoma: A case report on surgical and prosthetic management. Am J Case Rep. (2024) 25:e944651. doi: 10.12659/AJCR.944651 39321125. Published 2024 Sep 25. PMC11444523

[28] Beecher MB Tong JY Gilhotra A Selva D . Ameloblastic carcinoma with orbital invasion. Ophthalmic Plast Reconstr Surg. (2024) 40:e196–8. doi: 10.1097/IOP.0000000000002706 38722761

