# Supplementary material for: Ameloblastic carcinoma with isolated supramandibular lymph node metastasis in an adolescent: a case report and review of the literature
Source: Front Oncol. 2026 Sep 24;16:1899367. doi: 10.3389/fonc.2026.1899367 (PMC13645601; doi:10.3389/fonc.2026.1899367)
Supplement: Supplementary file 1 [file DataSheet1.pdf]

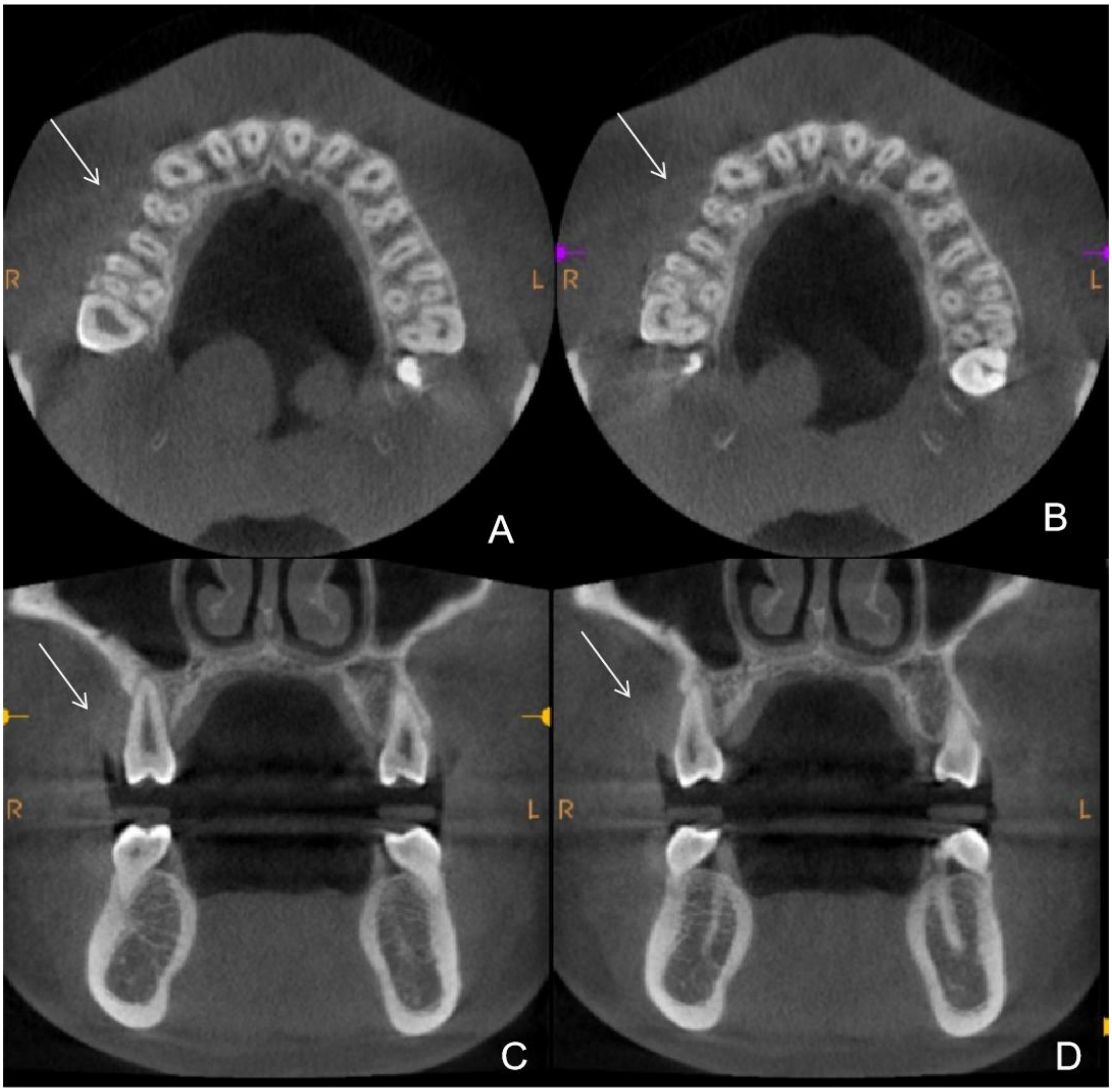

Figure S1 CBCT examination (13 December, 2023) (A.B) Axial views of CBCT examination. Buccal alveolar bone resorption is noted in the region of teeth 14 and 15. (C.D) coronal views of CBCT examination. It extends to the apical two thirds of the roots, without involvement of the maxillary sinus floor.

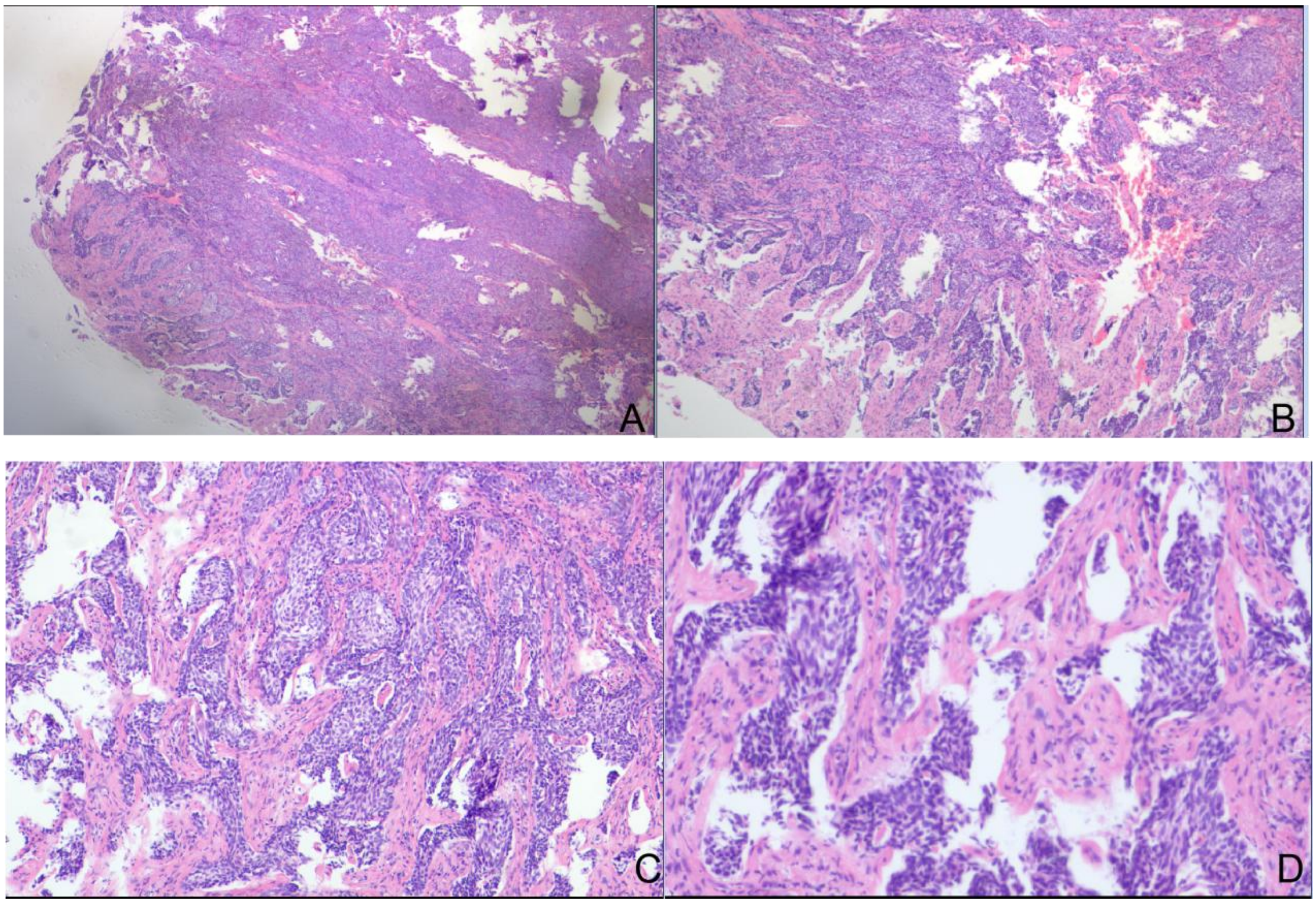

Figure S2 Light microscopic findings. (A)A solid tissue component with ill-defined borders is visualized, containing numerous scattered cell nests of varying sizes, as well as cord-like and sieve-like structures.(B)Dense epithelial-like cell nests enclose uniformly sized round or oval spaces. The tumor cell nuclei are small and hyperchromatic, with scant cytoplasm and closely packed cellular arrangement.(C)The regions surrounding the cystic spaces exhibit a bilayered structure: the inner layer comprises cuboidal/columnar epithelial-like cells, while the outer layer consists of basaloid cells with hyperchromatic nuclei and sparse cytoplasm. Mild to moderate nuclear atypia is present; nucleoli are not discernible, and occasional mitotic figures can be observed.(D)The tumor cells are small and polygonal/cuboidal, with round or oval, hyperchromatic nuclei and thickened nuclear membranes. Nucleoli are indistinct, and distinct basement membrane-like material is identifiable within the cystic spaces.(A) Hematoxylin-eosin (HE) stain, ×50.(B)HE, ×100.(C)HE, ×200.(D) HE, ×400

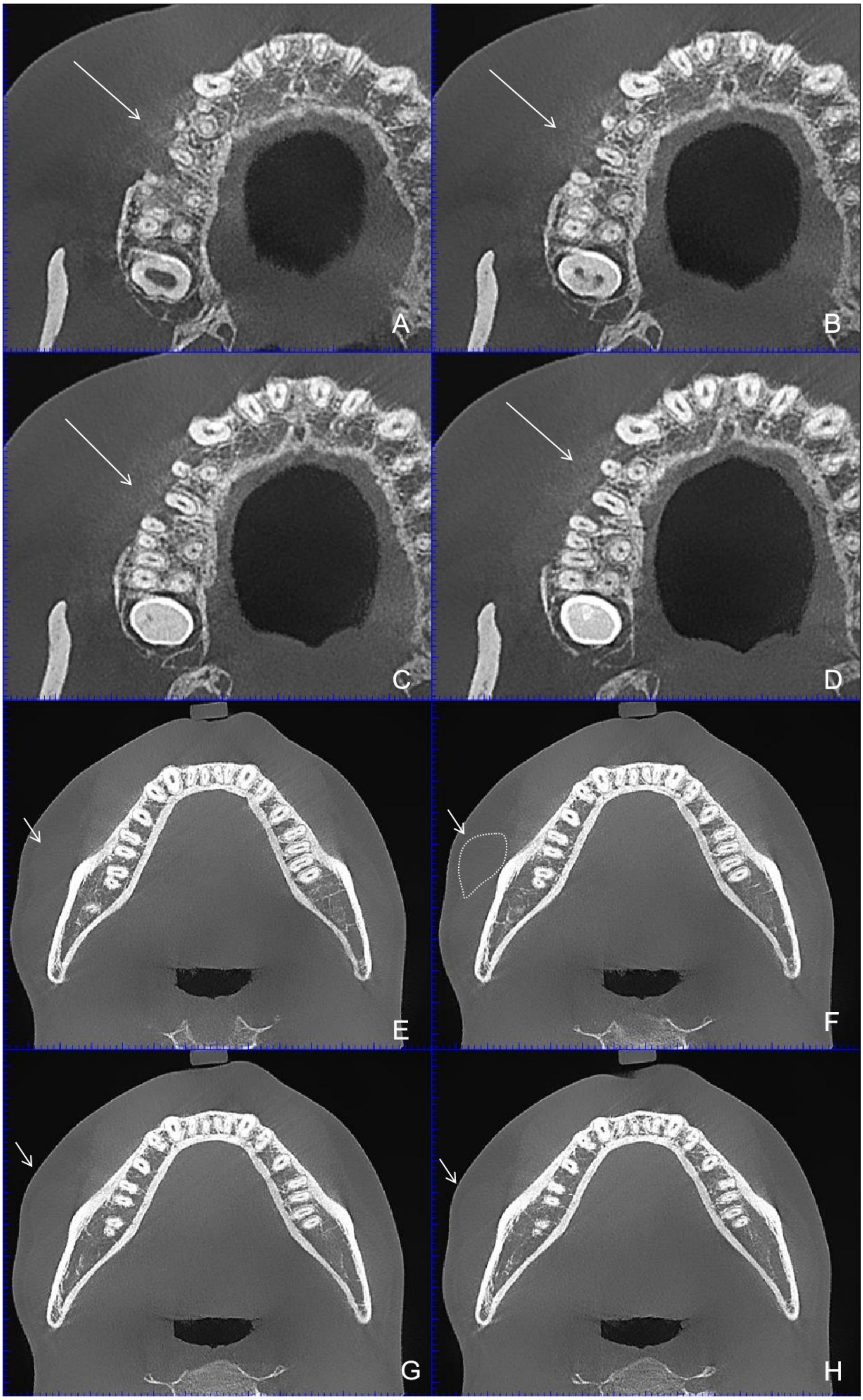

A
B
C
D
E
F
G
H

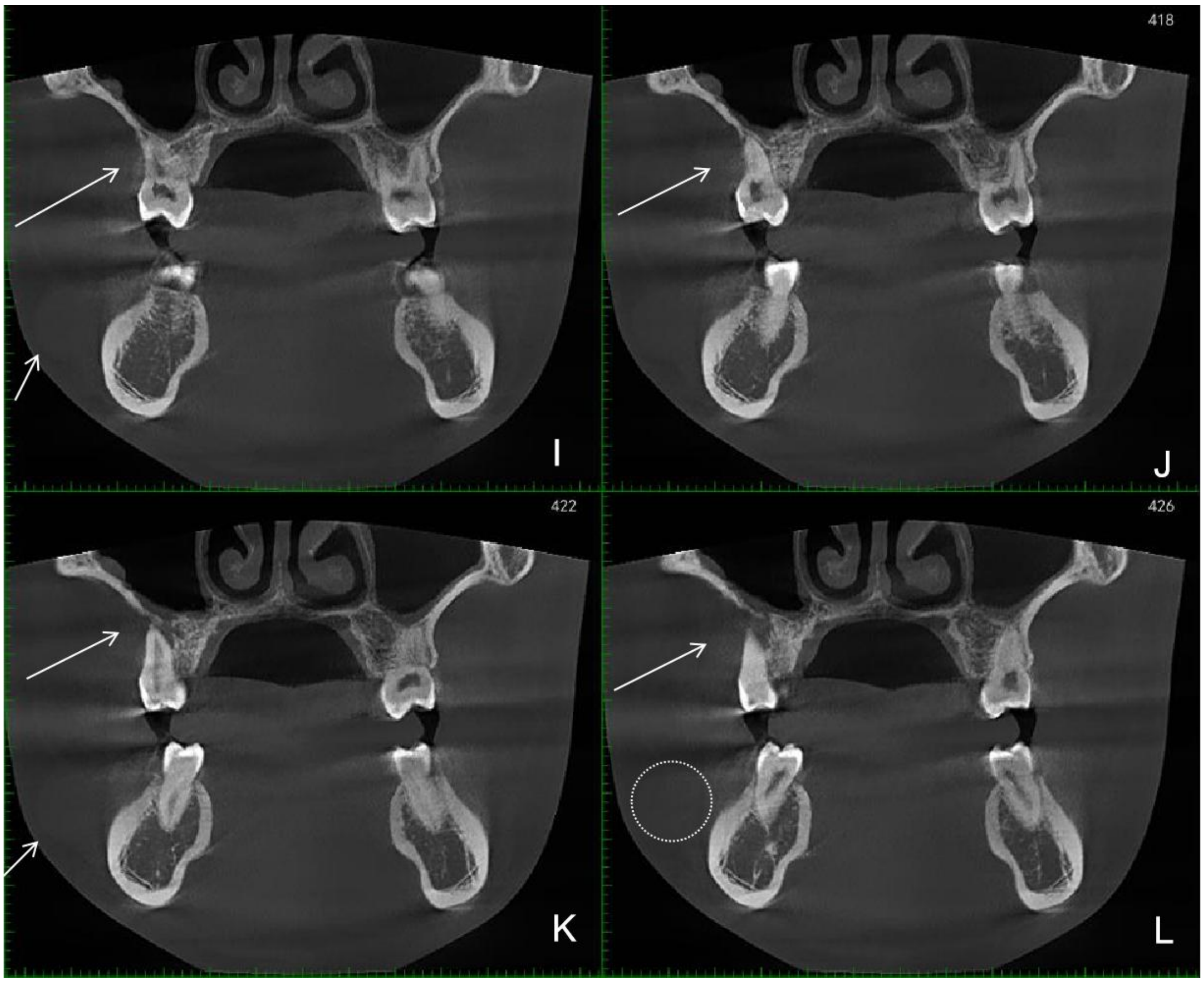

Figure S3 CBCT examination (17 May 2024) (A–H) Axial views of CBCT. (A–D) These images demonstrate further buccal alveolar bone resorption involving teeth 14 to 16, with an expanded resorption area. (E–H) A slight difference in gray level appears to be present within the lateral soft tissues of the right mandible, with dimensions measuring approximately 21 mm × 12 mm × 16 mm. (I–L) Coronal views of CBCT. Bone resorption has progressed to involve the entire root length of the affected teeth, extending to the root apices, and is accompanied by thinning of the maxillary sinus floor. The long arrow indicates the lesion in the right maxilla, and the short arrow indicates the lesion in the right mandible.

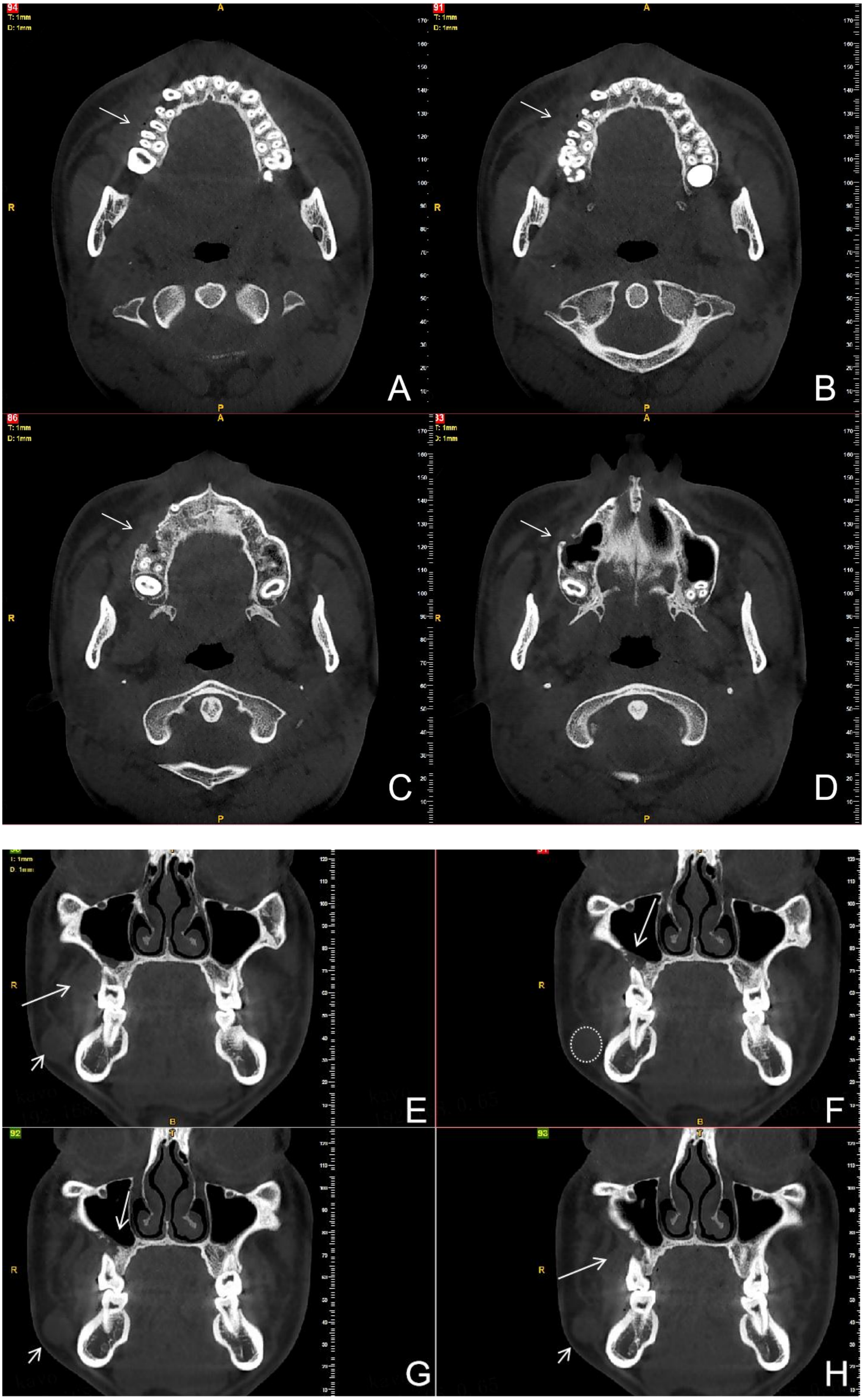
A
B
C
D
E
F
G
H

Figure S4 CBCT examination (15 July 2024) (A–D) Axial views of CBCT; (E–H) coronal views of CBCT. These images show buccal bone destruction and resorption involving teeth 13 to 17, accompanied by perforation of the maxillary sinus floor. A slight difference in gray level appears to be present within the soft tissue on the lateral side of the right mandible, with dimensions measuring approximately 21 mm × 12 mm × 16 mm. The long arrow indicates the lesion in the right maxilla, and the short arrow indicates the lesion in the right mandible.

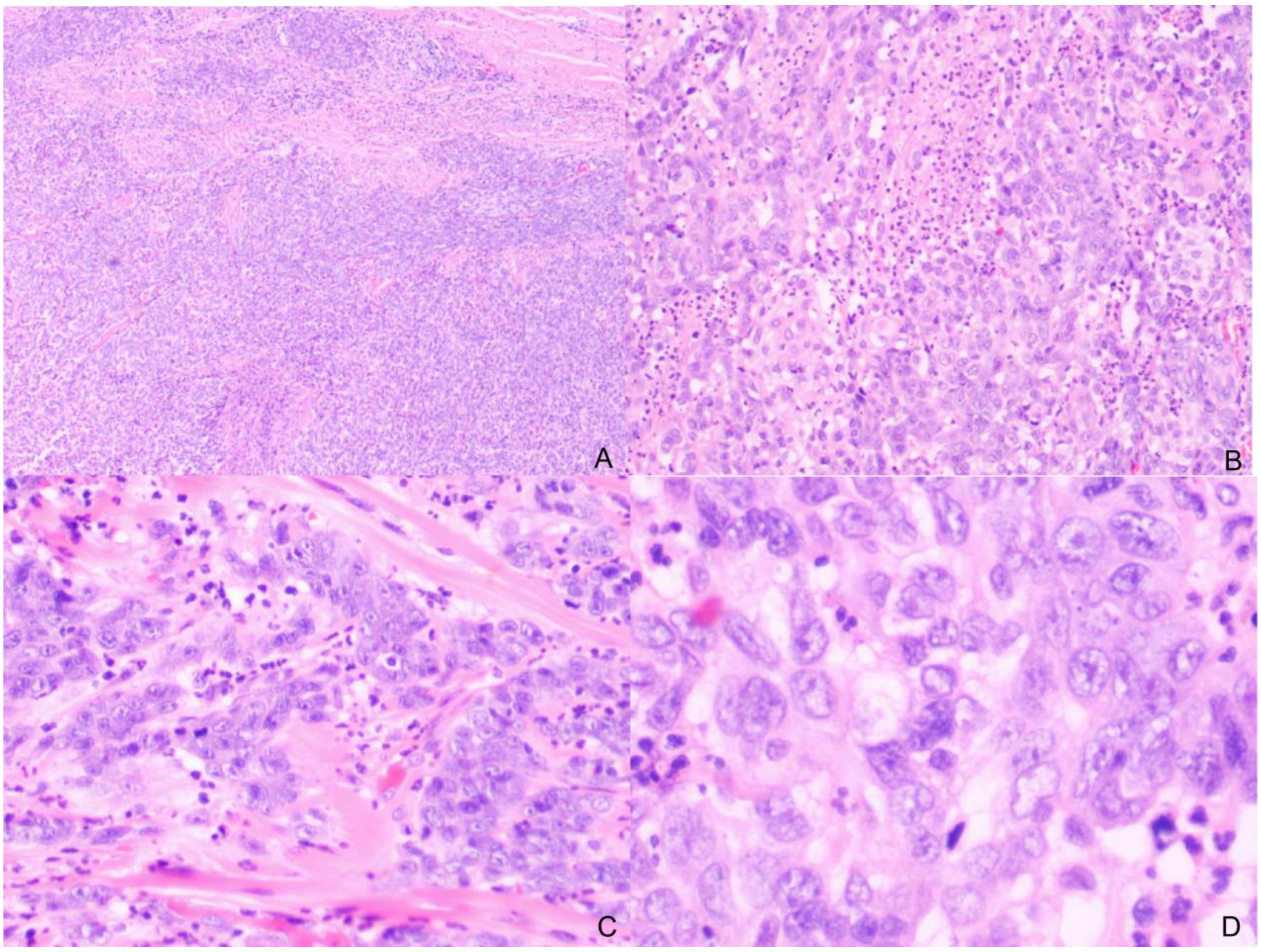

Figure S5 Light microscopic findings. Histopathological microscopy reveals diffuse nest-like proliferation of cancer cells. (A) A solid lesion is identified, characterized by diffuse sheet-like hyperplasia with infiltrative growth into the surrounding tissues. Irregular tumor nests and cord-like structures are observed invading adjacent tissues. Focal areas of necrosis and hemorrhage are present, indicative of rapid tumor progression. (B) The tumor cells are predominantly arranged in solid sheets, with focal follicular-like or microcystic structures. Cell borders are indistinct, and the cells exhibit nuclear pleomorphism, hyperchromasia, and coarsely granular chromatin. (C) The tumor cells show marked nuclear atypia, characterized by significant variation in nuclear size and irregular nuclear membranes. Residual trabecular bone-like structures are noted between tumor nests, suggestive of infiltrative osteoclastic growth. (D) Prominent nuclear pleomorphism, coarsely granular chromatin, and enlarged nucleoli are evident. Numerous pathological mitotic figures are identifiable, indicating high proliferative activity of the tumor cells. (A) Hematoxylin-eosin (HE) stain, ×40; (B) HE, ×100; (C) HE, ×200; (D) HE, ×400.

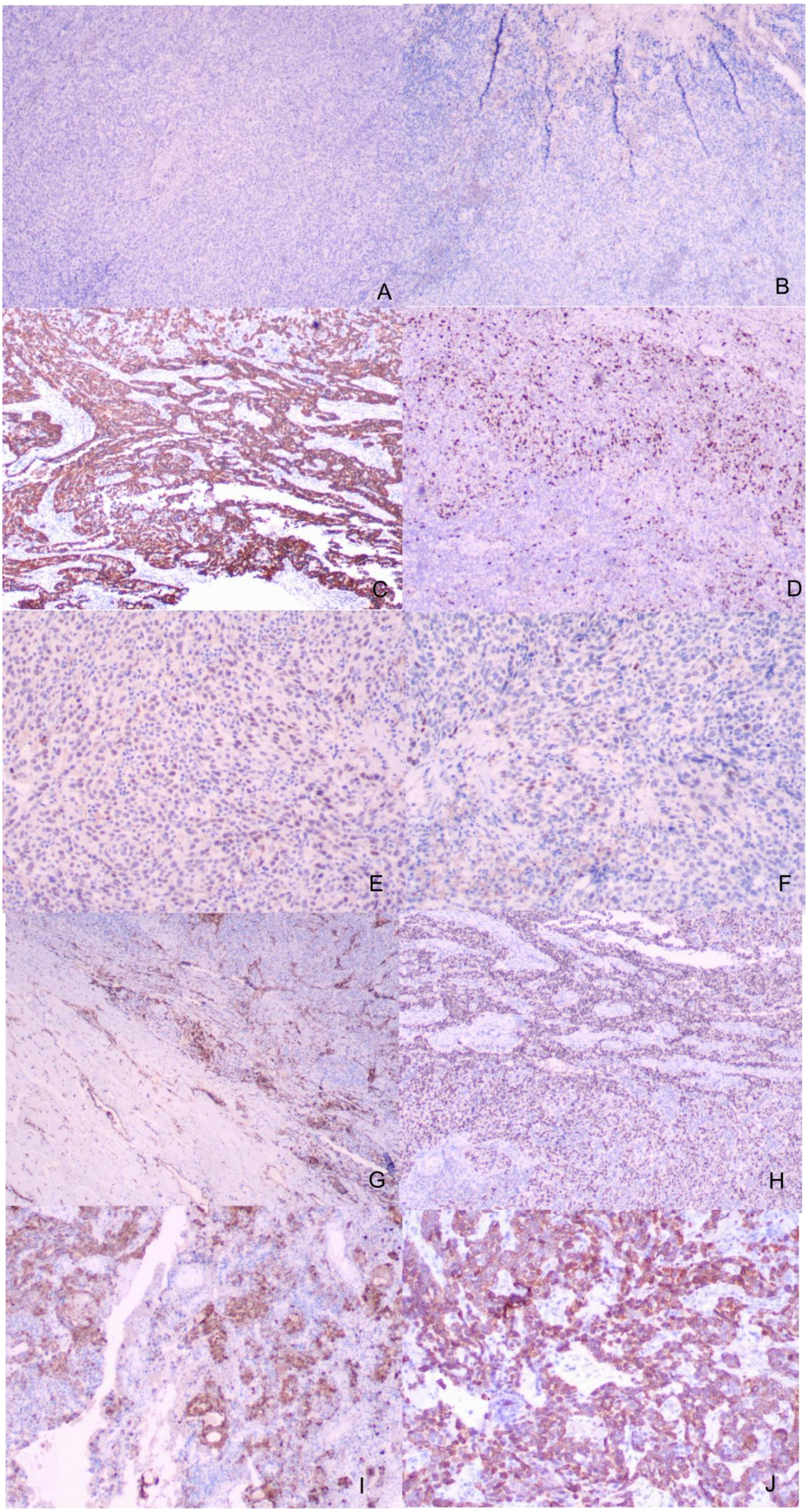
A
B
C
D
E
F
G
H
I
J

Figure S6 Immunohistochemistry (A) CK7: (-), (B) D2-40: (no lymphatic vessel cancer thrombus), (C) CK5/6: (+), (D) Ki-67: (positive cell count about 40%), (E) P40: (+), (F) P53: (positive cell count about 10%), (G) CD31: (no intravascular cancer thrombus), (H) P63: (+), (I) P16: (+), (J) CK19: (+) and the pathological diagnosis is AC

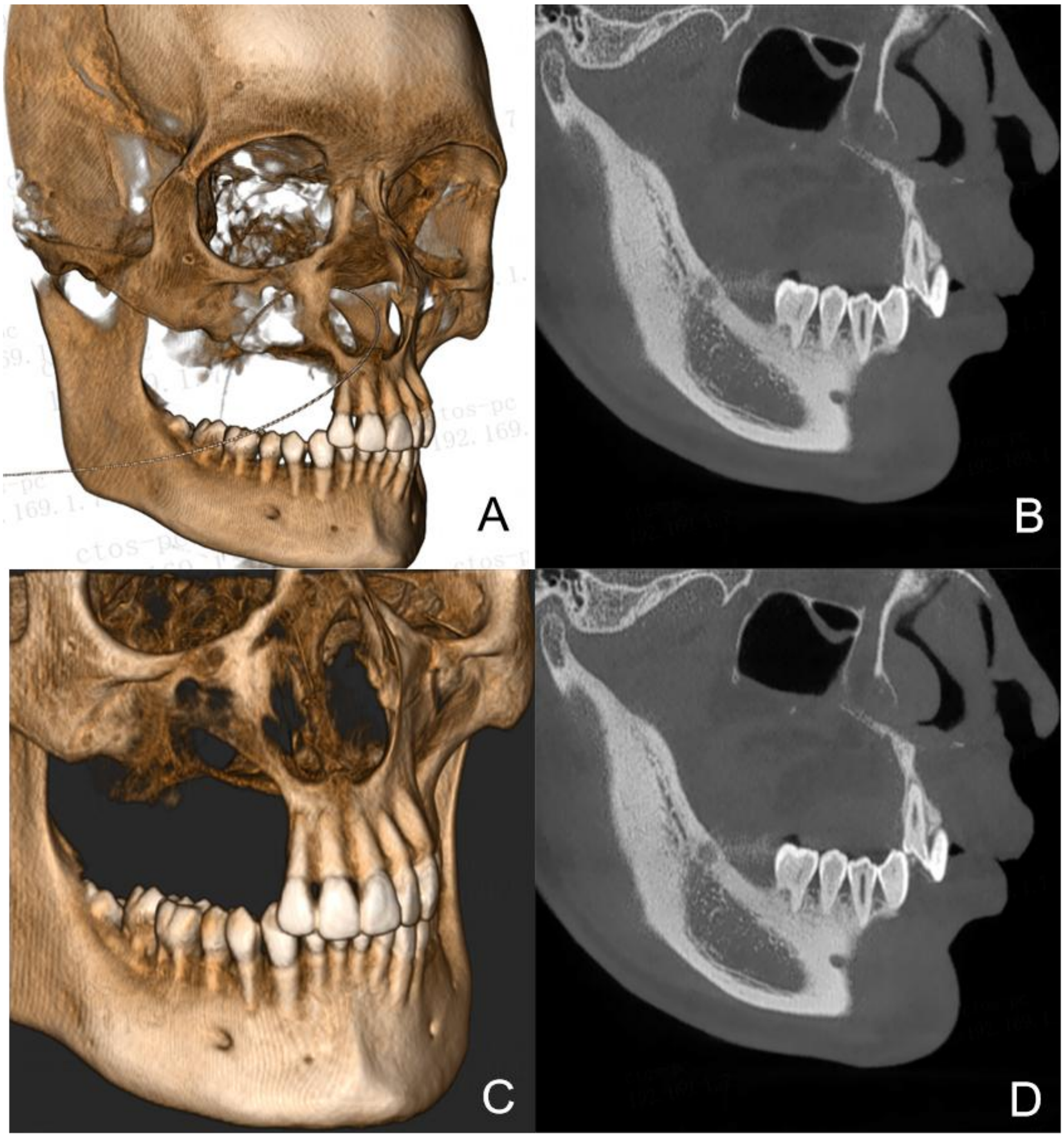

Figure S7 CBCT examination (A) Three-dimensional reconstruction and (B) oblique coronal view of CBCT examination (24 July 2024) show partial absence of the right maxilla and loss of teeth 13-17. (C) Three-dimensional reconstruction and (D) oblique coronal view of CBCT examination (17 November 2024) show no soft tissue mass or bone destruction shadow in the surgical area.

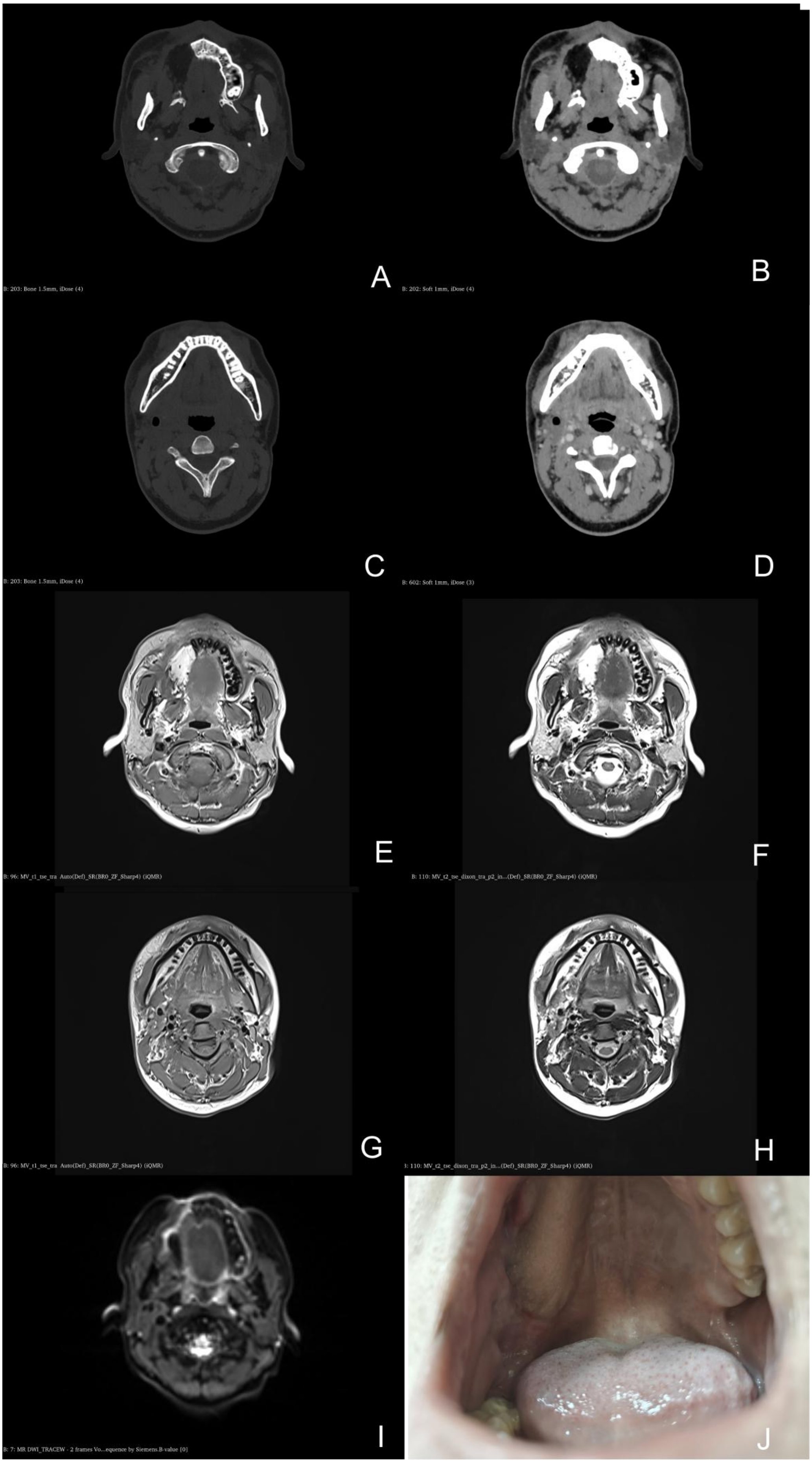
A
B: 203: Bone 1.5mm, iDose (4)
B
B: 202: Soft 1mm, iDose (4)
C
B: 203: Bone 1.5mm, iDose (4)
D
B: 602: Soft 1mm, iDose (3)
E
B: 96: MV_t1_tse_tra Auto(Def)_SR(BR0_ZF_Sharp4) (iQMR)
F
B: 110: MV_t2_tse_dixon_tra_p2_in...(Def)_SR(BR0_ZF_Sharp4) (iQMR)
G
B: 96: MV_t1_tse_tra Auto(Def)_SR(BR0_ZF_Sharp4) (iQMR)
H
I
B: 7: MR DWI_TRACEW - 2 frames Vo...equence by Siemens.B-value [0]
J

Figure S8 CT and MRI examination (22 September 2025). No tumor recurrence was observed in the surgical area.

(A-D) Axial views of CT; (E–I) Axial views of MRI. (J)The flap shows good survival and healing.

Table S1 Sequential progression throughout the entire disease course

Date	Clinical stage	Key clinical events	Core examination & diagnosis	Intervention & Outcome
13 Dec 2023	Initial presentation at external hospital	16-year-old male, gingival mass at 14/15, bleeding and tooth space widening	CBCT: buccal alveolar bone resorption; intraoperative frozen: ameloblastoma; final pathology: peripheral ameloblastoma	Resection of gingival mass + curettage of buccal bone plate
17 May 2024	Local recurrence & lesion progression	Right maxillary buccal/palatal gingival swelling, erosion	Serial CBCT: expanded bone destruction involving full root length, maxillary sinus floor thinning, occult soft tissue opacity lateral to right mandible	Referral to our hospital for radical surgery
15 Jul 2024	First admission to our hospital	Aggressive gingival erosion, tooth 14 mobility I°; no palpable cervical lymphadenopathy	CBCT: bone destruction with maxillary sinus floor perforation; preliminary clinical diagnosis: recurrent ameloblastoma	Extended tumorectomy + segmental maxillectomy (13–17 extraction) + submental island flap reconstruction
24 Jul 2024	Postoperative immediate evaluation	Postoperative recovery smooth	CBCT 3D reconstruction: partial right maxillary defect, complete resection of primary lesion	No residual bone lesion on imaging; discharge after wound healing
Jul 2024	Definitive malignant diagnosis	Postoperative histopathology + IHC	Diffuse carcinoma infiltration into bone marrow; Ki-67 ~40%, P40(+)/CK5/6(+)/CK7(-); final diagnosis: AC	Surgical margins negative; no adjuvant therapy arranged temporarily, scheduled regular follow-up
17 Nov 2024	Short-term follow-up (4 months post-first surgery)	Asymptomatic, normal flap appearance	CBCT: surgical site well-healed, no bone destruction or soft tissue mass detected	Continue routine outpatient surveillance
22 Mar 2025	Re-admission for metastatic lesion	Sudden enlargement of right buccal soft tissue mass (23×19×16 mm), painless, mobile lymph	Contrast-enhanced CT: heterogeneous mass with central necrosis adjacent to buccinator muscle; no mandibular bone	Preoperative judgment: suspected metastatic malignant tumor

Date	Clinical stage	Key clinical events	Core examination & diagnosis	Intervention & Outcome
		nodes in submandibular region	destruction	
Mar 2025	Second radical surgery	General anesthesia completed reoperation	Intraoperative frozen: malignant epithelial tumor; permanent pathology: AC with facial lymph node metastasis	Extended resection of buccal metastatic mass + right suprahyoid & mid-deep cervical lymph node dissection; 12 cervical nodes negative for metastasis
Mar–May 2025	Adjuvant chemoradiotherapy	Postoperative wound healed smoothly	No visible residual tumor on postoperative CT	Radiotherapy (total dose 60 Gy) + concurrent cisplatin chemotherapy (2-month course)
22 Sep 2025	Long-term follow-up (6 months post chemoradiotherapy)	Patient in good general condition, normal oral function, flap viable	Combined CT + MRI: no local recurrence, no pulmonary/distant metastasis; no enlarged cervical/facial lymph nodes	Long-term regular imaging surveillance planned for 3–5 years
